# Supplementary material for: Warming and altered precipitation independently and interactively suppress alpine soil microbial growth in a decadal-long experiment
Source: eLife. 2024 Apr 22;12:RP89392. doi: 10.7554/eLife.89392 (PMC11034942; doi:10.7554/eLife.89392)
Supplement: Source code 1. [file elife-89392-code1.zip › eLife-VOR-RA-2023-89392/Source Code 1.docx]

**Calculating taxon-specific changes in density**

Taxon-specific changes in density caused by isotope incorporation were calculated as shown in equations 1 to 12 below. We calculated the total number of 16S rRNA gene copies (*y_ijk_*) for bacterial taxon *i* in density fraction *k* of replicate *j* as follows:

$y_{ijk}$=$p_{ijk}$×$f_{jk}$ (1)

where *p_ijk_* is the relative abundance of each individual taxon (*i*) within an individual density fraction (*k*) from a particular replicate tube (*j*). *f_jk_* is the total number of 16S rRNA gene copies using the universal 16S rRNA primer for qPCR of each fraction (*k*) in each replicate density gradient (*j*).

The total number of 16S rRNA gene copies (*y_ij_*) for bacterial taxon (*i*) in replicate (*j*) is summed across all *K* density fractions as follows:

$y_{ij}$=$\sum_{1}^{K} y_{ijk}$ (2)

The density (*W_ij_*) for bacterial taxon (*i*) of replicate (*j*) was computed as a weighted average, summing across all *K* density fractions the density (*x_jk_*) of each individual fraction times the total number of 16S rRNA gene copies (*y_ijk_*) in that fraction, expressed as a proportion of the total 16S rRNA gene copies (*y_ij_*) for taxon (*i*) in replicate (*j*), as follows:

$W_{ij}$=$\sum_{1}^{K} x_{jk}$×$(\frac{y_{ijk}}{y_{ij}})$ (3)

For a given taxon (*i*), we calculated the difference in density caused by isotope incorporation (*Z_i_*) as follows:

$Z_{i}$=$W_{LABi}$×$W_{LIGHTi}$ (4)

where *W_LABi_* and *W_LIGHTi_* are the mean, across all replicates, of the ^18^O-labeled treatment and unlabeled treatment.

**Calculating taxon-specific GC content and molecular weight**

The GC content of each bacterial taxon (*G_i_*) was calculated using the mean density for the unlabeled (*W_LIGHTi_*) treatments. Below is the linear relationship between GC content (*G_i_*, expressed as a proportion) and unlabeled buoyant density (*W_LIGHTi_*) on a CsCl gradient (derived from pure cultures of microbial species with known and strongly differing GC contents) according to Hungate et al. (2015):

$G_{i}$=$\frac{1}{0.083506}$× ($W_{LIGHTi}-1.646057$) (5)

The natural abundance molecular weight of DNA is a function of GC content, based on the atomic composition of the four DNA nucleotides. Single-stranded DNA made of pure adenine (A) and thymine (T) has an average molecular weight of 307.691 g/mol. The corresponding average molecular weight for DNA comprising only guanine (G) and cytosine (C) is 308.187 g/mol. When theGCcontent is known, the average molecular weight of a single strand of DNA can be calculated using the following equation:

$M_{LIGHTi}$=$0.496G_{i}$+ 307.691 (6)

There are 12 oxygen atoms per DNA nucleotide pair, regardless of GC content: 6 each for G and C, 7 for T, and 5 for A. These atoms contain ^18^O at natural abundance, which we assume to be 0.002000429 atom fraction for ^18^O (Hungate et al. 2015). The maximum labeling is achieved when all oxygen atoms are replaced by ^18^O. Therefore, given the molecular weight of each additional neutron (1.008665 g/mol; Hungate et al. 2015), the maximal increase in molecular weight (corresponding to 1 atom fraction ^18^O, or 100% atom percent ^18^O) is 12.07747 g/mol. The theoretical maximum molecular weight (*M*_HEAVYMAX_*_i_*) of fully ^18^O -labeled DNA for taxon (*i*) is then calculated as follows:

$M_{\mathrm{HEAVYMAX}i}$= 12.07747 + $M_{\mathrm{LIGHT}i}$ (7)

However, as oxygen in DNA is derived from both water and organic sources, we also estimated the maximum molecular weight of DNA that could result from assimilation of ^18^O-water (*M*_HEAVY_*_i_*). This enables a more accurate estimate of growth, by accounting for the proportion of oxygen atoms in newly synthesized DNA derived from the labeled environmental water (*U*):

$M_{\mathrm{HEAVY}i}$= 12.07747*U* + $M_{\mathrm{LIGHT}i}$ (8)

*U* was estimated via sensitivity analysis, with logical lower and upper bounds. Values of *U* were rejected as too low if they resulted in a value for fully labeled DNA that was less than the molecular weight of labeled DNA observed in the 18O treatment (*M*_HEAVY_*_i_* < *M*_LAB_*_i_*), and values of U were rejected as too high if they resulted in estimates of unlabeled 16S rRNA gene copies at the end of the incubation that exceeded the measured abundance at the beginning of the incubation (*N*_LIGHT_*_it_* > *N*_LIGHT_*_i_*_0_), a violation of the assumption that all newly formed DNA in the 18O treatment was isotopically labeled. For all taxa, we used the empirical value of *U* (0.60) according to (Koch et al., 2018).

The molecular weight of DNA for taxon (*i*) in the labeled treatment (*M*_LAB_*_i_*) was calculated as follows:

$M_{\mathrm{LAB}i}$=$\left( \frac{Z_{i}}{W_{LIGHTi}}+1 \right)$× $M_{LIGHTi}$ (9)

The atom fraction excess of ^18^O for taxon (*i*) (*A*_OXYGEN_*_i_*), accounting for the background fractional abundance of ^18^O (0.002000429; Hungate et al. 2015), is then calculated as follows:

$A_{\mathrm{OXYGEN}i}$=$(\frac{M_{\mathrm{LAB}i}-M_{\mathrm{LIGHT}i}}{M_{\mathrm{HEAVYMAX}i}-M_{\mathrm{LIGHT}i}})$ × (1-0.002000429) (10)

**Modeling taxon-specific population growth**

To estimate taxon-specific bacterial growth, we used the model of ^18^O isotope substitution in DNA including an exponential model of population growth (Koch et al., 2018; Stone et al., 2021). We used a mixing model of DNA molecular weight to estimate abundances of unlabeled and labeled DNA fragments containing copies of 16S rRNA genes.

For each bacterial taxon (*i*), we assumed that the abundance of cells at time *t* was proportional to the abundance of 16S rRNA gene copies (*N*_TOTAL_*_i_*, with units of 16S rRNA gene copies/g soil). We further assumed that changes in bacterial abundances followed an exponential growth model over the 2-d incubation period, with the net rate of population growth (*r_i_*, units: d^-1^):

$N_{\mathrm{TOTAL}it}$=$N_{\mathrm{TOTAL}i0}$×$e^{r_{i}t}$ (11)

At time 0, the total abundance of 16S rRNA gene copies (*N*_TOTAL_*_i_*_0_) was equivalent to the abundance of unlabeled 16S rRNA gene copies at the beginning of the incubation (*N*_TOTAL_*_i_*_0_), but by the end of the incubation period, both unlabeled (*N*_LIGHT_*_it_*) and labeled (*N*_HEAVY_*_it_*) 16S rRNA gene copies may have been present such that:

$N_{\mathrm{TOTAL}it}$=$N_{\mathrm{LIGHT}it}$+ $N_{\mathrm{HEAVY}it}$ (12)

Taxon-specific abundances of 16S rRNA gene copies at the beginning (*N*_TOTAL_*_i_*_0_ = *N*_LIGHT_*_i_*_0_) and the end of the incubation (*N*_TOTAL_*_it_*) were calculated as the products of the total abundance of 16S rRNA gene copies across all taxa, determined by qPCR, and the relative abundance of 16S rRNA gene copies associated with taxon i, determined by sequencing. We used a linear mixing model of DNA molecular weights to estimate the abundance of labeled 16S rRNA gene copies at the end of the incubation (*N*_HEAVY_*_it_*), and by difference, the abundance of unlabeled 16S rRNA gene copies at the end of the incubation (*N*_LIGHT_*_it_*):

$N_{\mathrm{LIGHT}it}$=$N_{\mathrm{TOTAL}it}$×$(\frac{M_{\mathrm{HEAVY}i}-M_{\mathrm{LAB}i}}{M_{\mathrm{HEAVY}i}-M_{\mathrm{LIGHT}i}})$ (13)

where for each taxon (*i*), N_TOTAL_*_it_* is the total abundance of 16S rRNA gene copies at the end of the incubation, *M*_HEAVY_*_i_* is the molecular weight of ^18^O-labeled DNA, *M*_LIGHT_*_i_* is the molecular weight of unlabeled DNA in ^16^O treatment, and *M*_LAB_*_i_* is the average molecular weight of DNA at the end of the ^18^O-H_2_O incubation (i.e., ^18^O treatment).

Then, the growth rate of taxon (*i*) was calculated as:

*g_i_*=ln ($\frac{N_{\mathrm{TOTAL}it}}{N_{\mathrm{LIGHT}it}}$) ×$\frac{1}{t}$ (14)

where N_TOTAL_*_it_* is the number of total gene copies for taxon i and N_LIGHT_*_it_* represents the unlabeled 16S rRNA gene abundances of taxon (*i*) at the end of the incubation period (time *t*).

We further calculated the average growth rates (represented by the production of new16S rRNA gene copies of each taxon per g dry soil per day) along the incubation, using the following equation (Stone et al., 2021):

$\frac{dN_{i}}{dt}$=$N_{\mathrm{TOTAL}it}$ (1 -$e^{-g_{i}t}$) ×$\frac{1}{t}$ (15)

where *t* is the incubation time (day).

**Reference:**

Hungate, B., Mau, R., Schwartz, E., Caporaso, J., Dijkstra, P., Van Gestel, N. *et al.* (2015). *Quantitative microbial ecology through stable isotope probing*. Applied and Environmental Microbiology, 81, 7570-7581.

Koch, B., McHugh, T., Hayer, M., Schwartz, E., Blazewicz, S., Dijkstra, P. *et al.* (2018). Estimating taxon-specific population dynamics in diverse microbial communities. *Ecosphere*, 9, e02090.

Stone, B.W., Li, J., Koch, B.J., Blazewicz, S.J., Dijkstra, P., Hayer, M. *et al.* (2021). Nutrients cause consolidation of soil carbon flux to small proportion of bacterial community. *Nature Communications*, 12, 3381.

**###R code**

**##R 3.6.2**

rm(list=ls())

########### **Calculate EAF, birth rate, mortality rate, and net growth rate**######

library(phyloseq)

# load data in

setwd("E:\\1Knowledge\\R-run\\qSIP-WP-zOTUs\\ Growth rate calculation ")

sam1 <- read.table('data/samdata-WP.txt', sep='\t', header=T, row.names=1, comment.char='', check.names=F)

## Select one of them

##sam=sam1[which(sam1$day == "2"&sam1$fertilizer == "Y_cTcP"|sam1$day =="0"&sam1$fertilizer == "Y_cTcP"), ]##N0/1DAY

##sam=sam1[which(sam1$day == "2"&sam1$fertilizer == "Y_cT+P"|sam1$day =="0"&sam1$fertilizer == "Y_cT+P"), ]##N0/3DAY

##sam=sam1[which(sam1$day == "2"&sam1$fertilizer == "Y_cT-P"|sam1$day =="0"&sam1$fertilizer == "Y_cT-P"), ]##N0/6DAY

##sam=sam1[which(sam1$day == "2"&sam1$fertilizer == "Y_+TcP"|sam1$day =="0"&sam1$fertilizer == "Y_+TcP"), ]##N180/1DAY

##sam=sam1[which(sam1$day == "2"&sam1$fertilizer == "Y_+T+P"|sam1$day =="0"&sam1$fertilizer == "Y_+T+P"), ]##N180/3DAY

##sam=sam1[which(sam1$day == "2"&sam1$fertilizer == "Y_+T-P"|sam1$day =="0"&sam1$fertilizer == "Y_+T-P"), ]##N180/6DAY

###sam=sam1[which(sam1$day == "2"&sam1$fertilizer == "Y_W"|sam1$day =="0"&sam1$fertilizer == "Y_W"), ]##N360/1DAY

head(sam)

str(sam)

otu <- read.table('data/otutab_clean.txt', sep='\t', header=T, comment.char='', check.names=F)

rownames(otu) <- otu$`#OTU ID`

otu <- otu[,-1]

#

otu <- otu[rowSums(otu)/sum(otu)>=(0.005/100),]

otu=otu[,rownames(sam)]

otu=otu[which(rowSums(otu[,1:3])>0),]

otu <- as.matrix(otu)

otu[1:10,1:10]

tax <- read.table('data/taxonomy_clean.txt', sep='\t', header=T, comment.char='', check.names=F)

rownames(tax) <- tax$`OTUID`

tax <- tax[,-1]

tax=tax[rownames(otu),]

tax <- as.matrix(tax)

tax[1:5,1:5]

# combine into phyloseq data

dat <- phyloseq(otu_table(otu, taxa_are_rows=T),

tax_table(tax),

sample_data(sam))

# devtools::install_github('bramstone/qsip', ref='master')

library(qsip)

# specify your qSIP data

qsip_dat <- specify_qsip(dat,

density='density_g_ml',

abund='qpcr_abund',

rep_id='sample_id',

rep_group='fertilizer',

iso='18O',

iso_trt='isotope',

timepoint='day')

# specify frequency filtering criteria

qsip_dat@qsip@filter_levels <- create_filters(3, 4, soft=1)##

# atom excess fraction (AEF) or atom percent excess (APE),correction=F or T

qsip_dat1 <- calc_excess(qsip_dat,

separate_label=T,

filter=T,

correction=F)

atom_excess <- qsip_dat1@qsip[['atom_excess']]

write.csv(atom_excess,'process/AEF_Y_+T-P_2DAY.csv')

#Y_cTcP_2DAY / Y_cT+P_2DAY / Y_cT-P_2DAY / Y_+TcP_2DAY / Y_+T+P_2DAY / Y_+T-P_2DAY / Y_W_2DAY

# per-capita growth,correction=F or T

qsip_dat2 <- calc_pop(qsip_dat,

separate_label=T,

filter=T,

correction=F)

#death_rates <- qsip_dat2@qsip[['death_rate']]

#growth_rates <- qsip_dat2@qsip[['growth_rate']]

birth_rates <- qsip_dat2@qsip[['birth_rate']]

#write.csv(death_rates,'process/death_rates_Y_cTcP_2DAY.csv')########

#write.csv(growth_rates,'process/growth_rates_Y_W_2DAY.csv')

write.csv(birth_rates,'process/birth_rates_Y_+T-P_2DAY.csv')

#Y_cTcP_2DAY / Y_cT+P_2DAY / Y_cT-P_2DAY / Y_+TcP_2DAY / Y_+T+P_2DAY / Y_+T-P_2DAY / Y_W_2DAY

####

########## Calculate confidence intervals ####

library(phyloseq)

# load data in

setwd("E:\\1Knowledge\\R-run\\qSIP-WP-zOTUs\\ Growth rate calculation ")

library(qsip)

library(boot)

set.seed(1234)

##choose one #Y_cTcP_2DAY / Y_cT+P_2DAY / Y_cT-P_2DAY / Y_+TcP_2DAY / Y_+T+P_2DAY / Y_+T-P_2DAY

#atom_excess=read.csv("process/AEF_Y_+T-P_2DAY.csv",header=T,row.names=1)

N=atom_excess

N=as.matrix(N)

#birth_rates=read.csv("process/birth_rates_Y_+T-P_2DAY.csv",header=T,row.names=1)

B=birth_rates

N=as.matrix(B)

head(N)

###############

N =na.omit(N)

M=as.data.frame.array(N)

G=as.data.frame(matrix(1:nrow(N),nrow=nrow(N),ncol=3))

for (i in 1:(nrow(N)))

{

X=N[i,]

b <- boot(X, function(u,i) mean(u[i]), R = 1000)

CI=boot.ci(b, index=1)#,type = c("norm", "basic", "perc")

CL <- CI$normal[2]

CU <- CI$normal[3]

G[i,c("V1")]=mean(N[i,])

G[i,c("V2")] =CL

G[i,c("V3")] =CU

}

P=cbind(M,G)

library(tidyverse)#install.packages("tidyverse")

P=plyr::rename(P, c("V1"="mean","V2"="95CL","V3"="95CU"))

###################

write.csv(P,'result/conf_EAF_Y_+T-P_2DAY.csv')

write.csv(P,'result/conf_birth_rates_Y_+T-P_2DAY.csv')

#Y_cTcP_2DAY / Y_cT+P_2DAY / Y_cT-P_2DAY / Y_+TcP_2DAY / Y_+T+P_2DAY / Y_+T-P_2DAY / Y_W_2DAY

##

########### EAF plot#################

##OTU_level

###Excess atom fraction 18O

library(ggnewscale)

library(ggplot2)

windowsFonts(myFont=windowsFont("Arial"))#

###

spe<-read.csv("result/conf_EAF_Y_cTcP_2DAY.csv",header=T,row.names = 1,check.names=F)

#Y_cTcP_2DAY / Y_cT+P_2DAY / Y_cT-P_2DAY / Y_+TcP_2DAY / Y_+T+P_2DAY / Y_+T-P_2DAY / Y_W_2DAY

head(spe)

tax <- read.table('clearing_data/taxonomy_clean.txt', sep='\t', header=T, comment.char='', check.names=F)

rownames(tax) <- tax$`OTUID`

tax <- tax[,-1]

tax=tax[rownames(spe),]

tax[1:5,1:5]

pro=tax[which(tax$Phylum=="Proteobacteria"),]

pro2=pro[,c(3,7)]

names(pro2)<-c("Phylum","Species")

pro2=as.data.frame(pro2)

unpro=tax[which(tax$Phylum!="Proteobacteria"),]

unpro2=unpro[,c(2,7)]

unpro2=as.data.frame(unpro2)

new_phy=rbind(pro2,unpro2)

names(new_phy)<-c("Phylum1","Species")

head(new_phy)

imp=merge(tax[,c(1:2)],new_phy,by="row.names",all=T)

rownames(imp)=imp$Row.names

imp=merge(imp[,c(3:4)],spe,by="row.names",all=T)

rownames(imp)=imp$Row.names

imp$group = ifelse(imp$`95CL` <= "0","B","A")

imp=as.data.frame.array(imp)

#imp=imp[order(imp$Phylum,imp$Phylum1,imp$mean,decreasing = F),]

imp=imp[order(imp$Phylum1,imp$mean,decreasing = F),]

for (i in 1:(nrow(imp)-1))

{imp[i,]$Row.names = ifelse(as.data.frame.array(imp[i,])$Phylum1 !=

as.data.frame.array(imp[i+1,])$Phylum1,

as.data.frame.array(imp[i,])$Phylum1, " ")}

imp[nrow(imp),]$Row.names=as.data.frame.array(imp[nrow(imp),])$Phylum1

head(imp)

##

imp=imp[-which(imp$Phylum1=="Unassigned"),]

N=c(1:nrow(imp))

imp1=cbind(imp,N)

for (i in 1:(nrow(imp1)))

{imp1[i,]$N = ifelse(imp1[i,]$Row.names == " ",5*i, 5*i+5)}

head(imp1)

spe=imp1

library(dplyr)

spe=rename(spe,'CL'=`95CL`)

spe=rename(spe,'CU'=`95CU`)

pd <- position_dodge(0.5)

A=spe[spe$group=="A",]

B=spe[spe$group=="B",]

p9<-ggplot(spe, aes(x=mean, y=N))+

geom_point(data=B,position=pd, size=2, shape=21,aes(colour=Phylum1,fill=group))+

scale_fill_manual(values =alpha(("black"),0.4))+

scale_color_manual(values=c("Acidobacteria"="#EE6677","Actinobacteria"="#00FF3B","Alphaproteobacteria"="#882E72","Armatimonadetes"="#5289C7","Bacteroidetes"="#DC050C","Betaproteobacteria"="#994F88","candidate_division_WPS-1"="#90C987","Chloroflexi"="#EE8026","Deltaproteobacteria"="#BA8DB4","Firmicutes"="#66CCEE","Gammaproteobacteria"="#AA6F9E","Gemmatimonadetes"="#0009AA","Ignavibacteriae"="#BBCCEE","Nitrospirae"="#F7F056","Planctomycetes"="#662506","Unassigned"="#7C7C7C","Verrucomicrobia"="#FCF7D5","Cyanobacteria"="#9E8B1E","Aminicenantes"="#420073","Latescibacteria"="#1B7837","Acetothermia"="#3C3A8D"))+

new_scale_fill() +#

new_scale_color() +###

geom_point(data=A,position=pd, size=2, shape=21, colour="black",aes(fill=Phylum1))+

scale_fill_manual(values=c("Acidobacteria"="#EE6677","Actinobacteria"="#00FF3B","Alphaproteobacteria"="#882E72","Armatimonadetes"="#5289C7","Bacteroidetes"="#DC050C","Betaproteobacteria"="#994F88","candidate_division_WPS-1"="#90C987","Chloroflexi"="#EE8026","Deltaproteobacteria"="#BA8DB4","Firmicutes"="#66CCEE","Gammaproteobacteria"="#AA6F9E","Gemmatimonadetes"="#0009AA","Ignavibacteriae"="#BBCCEE","Nitrospirae"="#F7F056","Planctomycetes"="#662506","Unassigned"="#7C7C7C","Verrucomicrobia"="#FCF7D5","Cyanobacteria"="#9E8B1E","Aminicenantes"="#420073","Latescibacteria"="#1B7837","Acetothermia"="#3C3A8D"))+

geom_errorbarh(data=B,aes(xmin=CL, xmax=CU,alpha=0.5), height=0, position=pd,size=1,colour="grey")+

geom_errorbarh(data=A,aes(xmin=CL, xmax=CU,colour=Phylum1,alpha=0.5), height=0, position=pd,size=1)+

scale_colour_manual(values=c("Acidobacteria"="#EE6677","Actinobacteria"="#00FF3B","Alphaproteobacteria"="#882E72","Armatimonadetes"="#5289C7","Bacteroidetes"="#DC050C","Betaproteobacteria"="#994F88","candidate_division_WPS-1"="#90C987","Chloroflexi"="#EE8026","Deltaproteobacteria"="#BA8DB4","Firmicutes"="#66CCEE","Gammaproteobacteria"="#AA6F9E","Gemmatimonadetes"="#0009AA","Ignavibacteriae"="#BBCCEE","Nitrospirae"="#F7F056","Planctomycetes"="#662506","Unassigned"="#7C7C7C","Verrucomicrobia"="#FCF7D5","Cyanobacteria"="#9E8B1E","Aminicenantes"="#420073","Latescibacteria"="#1B7837","Acetothermia"="#3C3A8D"))+

theme_bw()+

theme(legend.position="none")+

theme(panel.grid.major = element_blank(),

panel.grid.minor = element_blank(),

panel.border = element_blank(),

axis.line=element_line(colour="black",size=1))+

theme(axis.text.y = element_blank(),axis.ticks.y = element_blank())+

theme(axis.text.x= element_text(size=12, family="myFont", color="black", face= "bold.italic", vjust=0.5, hjust=0.5),

element_line(colour="black",size=1))+

geom_vline(aes(xintercept=0),linetype="dashed")+

#xlim(-0.35,0.5)+

xlab("Excess atom fraction 18O") +

ylab("Ranked OTUs") +#

ggtitle("Y_cTcP_2DAY")+#Y_cTcP_2DAY / Y_cT+P_2DAY / Y_cT-P_2DAY / Y_+TcP_2DAY / Y_+T+P_2DAY / Y_+T-P_2DAY / Y_W_2DAY

theme(title= element_text(size=15, family="myFont", color="black", face= "bold", vjust=0.5, hjust=0.5))+

theme(plot.title = element_text(hjust = 0.5))+

geom_text(aes(label = Row.names,colour=Phylum1,x = 0.2))

p9

ggsave("./Fig/EAF_Y_cTcP_2DAY.pdf",device=cairo_pdf,width=4,height=4)

#Y_cTcP_2DAY / Y_cT+P_2DAY / Y_cT-P_2DAY / Y_+TcP_2DAY / Y_+T+P_2DAY / Y_+T-P_2DAY / Y_W_2DAY

######

library(ggpubr)

ggarrange(p7,p8,p9,ncol=3,nrow=1)

ggsave("./Fig/EAF_N360.pdf",device=cairo_pdf,width=8,height=4)

ggarrange(p1,p2,p3,p4,p5,p6,p7,ncol=3,nrow=3)

ggsave("./Fig/EAF_All.pdf",device=cairo_pdf,width=16,height=12)

####

########### Draw a chart of birth rate/mortality rate/net growth rate ####

rm(list=ls())

library("ggplot2")

windowsFonts(myFont=windowsFont("Arial"))

####

birth<-read.csv("result/Y_W/conf_birth_rates_Y_W_2DAY.csv",header=T,row.names = 1,check.names=F)

death<-read.csv("result/Y_W/conf_death_rates_Y_W_2DAY.csv",header=T,row.names = 1,check.names=F)

growth<-read.csv("result/Y_W/conf_growth_rates_Y_W_2DAY.csv",header=T,row.names = 1,check.names=F)

birth$OTU=rownames(birth)

death$OTU=rownames(death)

growth$OTU=rownames(growth)

birth$group="birth_rates"

death$group="death_rates"

growth$group="growth_rates"

growth=growth[order(growth$mean,decreasing =T),]

N=c(1:nrow(growth))

growth=cbind(growth,N)

birth=merge(birth,growth[,c(which(colnames(growth)=="OTU"),which(colnames(growth)=="N"))],by.x="OTU",all=F)

death=merge(death,growth[,c(which(colnames(growth)=="OTU"),which(colnames(growth)=="N"))],by.x="OTU",all=F)

aa=rbind(birth,death,growth)

aa=rename(aa,'CL'=`95CL`)

aa=rename(aa,'CU'=`95CU`)

head(aa)

p14<-ggplot(data=aa, aes(x=N, y=mean, group=group, shape=group,colour=group)) +

geom_errorbar(aes(ymin=CL,ymax=CU),width=.001,size=0.5)+

geom_line(size=0.7) +

geom_point(size=1.2, fill="white") +

scale_shape_manual(values=c(21,21,21))+

scale_colour_manual(values=c("birth_rates"="#00bfc4","death_rates"="#e83631","growth_rates"="#3A398C"))+

theme_bw() +

theme(axis.text.x = element_blank(),axis.ticks.x = element_blank())+

theme(axis.text.y= element_text(size=12, family="myFont", color="black", face= "bold.italic", vjust=0.5, hjust=0.5),

element_line(colour="black",size=1))+

theme(legend.position="none")+

theme(panel.grid.major = element_blank(),

panel.grid.minor = element_blank(),

panel.border = element_blank(),

axis.line=element_line(colour="black",size=0.8))+

theme(legend.position="none")+

geom_hline(aes(yintercept=0),linetype="dashed",color="red")+

xlab("Ranked OTUs") +

ylab("r (day−1)") +

theme(axis.text= element_text(size=12, family="myFont", color="black", face= "bold", vjust=0.5, hjust=0.5),

element_line(colour="black",size=1))+

ggtitle("Y_W_2DAY")+

theme(title= element_text(size=15, family="myFont", color="black", face= "bold", vjust=0.5, hjust=0.5))+

theme(plot.title = element_text(hjust = 0.5))+

ylim(-2,1)+##

theme(legend.justification=c(1,1),#

legend.position="bottom")#

p14

##ggsave("Fig/rate_Y_W_2DAY.pdf",device=cairo_pdf,width=4,height=4)

#p8=cTcP,p9=cT+P,p10=cT-P,p11=+TcP,p12=+T+P,p13=+T-P,p14=W

######

library(ggpubr)

ggarrange(p7,p8,p9,ncol=3,nrow=1)

ggsave("./Fig/rate_Y_cTcP_2DAY.pdf",device=cairo_pdf,width=8,height=3)

ggarrange(p8,p9,p10,p11,p12,p13,p14,ncol=3,nrow=3)

ggsave("./Fig/rate_All.pdf",device=cairo_pdf,width=16,height=8)

####

############ Stacked cumulative graph of growth and death ###########

rm(list=ls())

sam1 <- read.table('clearing_data/samdata-WP.txt', sep='\t', header=T, row.names=1, comment.char='', check.names=F)

##

sam=sam1[which(sam1$day =="0"&sam1$fertilizer == "Y_cTcP"), ]##

head(sam)

#

otu <- read.table('clearing_data/otutab_clean.txt', sep='\t', header=T, comment.char='', check.names=F)

rownames(otu) <- otu$`#OTU ID`

otu <- otu[,-1]

#

otu <- otu[rowSums(otu)/sum(otu)>=(0.005/100),]

otu=otu[,rownames(sam)]

otu=otu[which(rowSums(otu[,1:3])>0),]

otu<-as.matrix(otu)

library("questionr")

otu1 <- cprop(otu)

rel_otu=as.data.frame.array(otu1[!rownames(otu1) %in% c("Total") , -which(colnames(otu1) %in% c("All"))])

head(rel_otu)

##

tax <- read.table('clearing_data/taxonomy_clean.txt', sep='\t', header=T, comment.char='', check.names=F)

rownames(tax) <- tax$`OTUID`

tax <- tax[,-1]

tax=tax[rownames(rel_otu),]

tax[1:5,1:5]

pro=tax[which(tax$Phylum=="Proteobacteria"),]

pro2=pro[,c(3,7)]

names(pro2)<-c("Phylum","Species")

pro2=as.data.frame(pro2)

unpro=tax[which(tax$Phylum!="Proteobacteria"),]

unpro2=unpro[,c(2,7)]

unpro2=as.data.frame(unpro2)

new_phy=rbind(pro2,unpro2)

names(new_phy)<-c("Phylum1","Species")

head(new_phy)

imp=merge(tax[,c(1:2)],new_phy,by="row.names",all=T)

rownames(imp)=imp$Row.names

tax_otu=merge(imp[,c(3:4)],rel_otu,by="row.names",all=F)

rownames(tax_otu)=tax_otu$Row.names

head(tax_otu)

tax_otu=as.data.frame.array(tax_otu)

##

tax_otu[,4]=tax_otu[,4]*sam[1,2]

tax_otu[,5]=tax_otu[,5]*sam[2,2]

tax_otu[,6]=tax_otu[,6]*sam[3,2]

###birth_rates/death_rates/growth_rates

a<-read.csv("result/Y_cTcP/conf_birth_rates_Y_cTcP_2DAY.csv",header=T,row.names = 1,check.names=F)

b<-read.csv("result/Y_cTcP/conf_death_rates_Y_cTcP_2DAY.csv",header=T,row.names = 1,check.names=F)

c<-read.csv("result/Y_cTcP/conf_growth_rates_Y_cTcP_2DAY.csv",header=T,row.names = 1,check.names=F)

a$OTU=rownames(a)

b$OTU=rownames(b)

c$OTU=rownames(c)

d=merge(a[,c(1:3,which(colnames(a)=="OTU"))],b[,c(1:3,which(colnames(b)=="OTU"))],by="OTU",all=T)

rate=merge(d,c[,c(1:3,which(colnames(c)=="OTU"))],by="OTU",all=T)

colnames(rate)=c("OTU","day1_1","day1_2","day1_3","day3_1","day3_2","day3_3","day6_1","day6_2","day6_3")

rownames(rate)=rate$OTU

rate=rate[,-1]

head(rate)

Qrate=merge(rate,tax_otu,by="row.names",all=F)

Qrate[,2]=exp(Qrate[,2]*2)*Qrate[,14]/2

Qrate[,3]=exp(Qrate[,3]*2)*Qrate[,15]/2

Qrate[,4]=exp(Qrate[,4]*2)*Qrate[,16]/2

Qrate[,5]=exp(Qrate[,5]*2)*Qrate[,14]/2

Qrate[,6]=exp(Qrate[,6]*2)*Qrate[,15]/2

Qrate[,7]=exp(Qrate[,7]*2)*Qrate[,16]/2

Qrate[,8]=exp(Qrate[,8]*2)*Qrate[,14]/2

Qrate[,9]=exp(Qrate[,9]*2)*Qrate[,15]/2

Qrate[,10]=exp(Qrate[,10]*2)*Qrate[,16]/2

Qrate[is.na(Qrate)] <- 0

head(Qrate)

Phylum=aggregate(Qrate[,c(2:10)],list(Qrate$Phylum1),sum)

rownames(Phylum)=Phylum$Group.1

Phylum=Phylum[,-1]

##

Phylum$day1=rowMeans(Phylum[,1:3])

Phylum$day3=rowMeans(Phylum[,4:6])

Phylum$day6=rowMeans(Phylum[,7:9])

Phylum=Phylum[,10:12]

Phylum = Phylum[(order(-rowSums(Phylum[,]))), ]

#other = colSums(Phylum[1:(dim(Phylum)[1]-11),])

#Phylum = Phylum[(dim(Phylum)[1]-10):dim(Phylum)[1], ]

#Phylum = rbind(Phylum,other)

#rownames(Phylum)[12] = c("Low abundance")

Phylum$tax=rownames(Phylum)

library(reshape2)

Phylum = as.data.frame(melt(Phylum, id.vars=c("tax")))

write.csv(Phylum, "Phylum-CW.csv")

Phylum1= read.csv("Phylum-zong-death.csv",header=T, row.names=1)

Phylum1

library(ggplot2)

p = ggplot(Phylum, aes(x=variable, y = value, fill = tax )) +

geom_bar(stat = "identity",position="stack", width=1)+

theme(strip.background = element_blank())+

theme_bw() +

xlab("")+ylab("growth rate (16S rRNA gene copies/d)")+

ggtitle("N360")+

#theme_classic()+theme(axis.text.x=element_text(angle=45,vjust=1, hjust=1))+

scale_fill_manual(values =rev(c("Acidobacteria"="#EE6677","Actinobacteria"="#00FF3B","Alphaproteobacteria"="#882E72","Armatimonadetes"="#5289C7","Bacteroidetes"="#DC050C","Betaproteobacteria"="#994F88","candidate_division_WPS-1"="#90C987","Chloroflexi"="#EE8026","Deltaproteobacteria"="#BA8DB4","Firmicutes"="#66CCEE","Gammaproteobacteria"="#AA6F9E","Gemmatimonadetes"="#0009AA","Ignavibacteriae"="#BBCCEE","Nitrospirae"="#F7F056","Planctomycetes"="#662506","Unassigned"="#7C7C7C","Verrucomicrobia"="#FCF7D5","Cyanobacteria"="#9E8B1E","Aminicenantes"="#420073","Latescibacteria"="#1B7837","Acetothermia"="#3C3A8D"))) +

theme(panel.grid = element_blank(), panel.background = element_rect(color = 'black', fill = 'transparent')) +

theme(legend.title = element_blank())

p

ggsave("./Fig/Copy_death_Y_cTcP_2DAY.pdf",device=cairo_pdf,width=8,height=8)

**###Calculation of the average growth rates (represented by the production of new16S rRNA gene copies of each taxon per g dry soil per day)**

setwd("E:\\1Knowledge\\R-run\\qSIP-WP-zOTUs\\ Growth rate calculation \\3 merged birth tables ")

rm(list=ls())

BD=read.csv("birthabove0anddeath1_+T-P.csv",header=T,row.names=1)#########

BD1=BD[BD$b95CL.x>0&BD$c95CL.y<0,]

str(BD)

str(BD1)

B=BD1[,1:4]

head(B)

sam1 <- read.table('../data/samdata-WP.txt', sep='\t', header=T, row.names=1, comment.char='', check.names=F)

sam=sam1[which(sam1$day == "2"&sam1$fertilizer == "Y_+T-P"&sam1$isotope =="18O"), ]###############################################################################

head(sam)

#str(sam)

otu <- read.table('../data/otutab_clean.txt', sep='\t', header=T, comment.char='', check.names=F)

rownames(otu) <- otu$`#OTU ID`

otu <- otu[,-1]

otu=otu[,rownames(sam)]#head(otu[,rownames(sam)])

#otu=otu[which(rowSums(otu)>0),]

otu <- as.matrix(otu)

otu[1:10,1:10]

#

library("questionr")

otu1 <- cprop(otu)

rel_otu=as.data.frame.array(otu1[!rownames(otu1) %in% c("Total") , -which(colnames(otu1) %in% c("All"))])#

#head(rel_otu)

#head(otu1)

#write.csv(rel_otu,"rel_otu.csv")

#write.csv(otu1,"otu1.csv")

rel_otu1=rel_otu/100

#write.csv(rel_otu1,"rel_otu1.csv")

#head(rel_otu1)

#str(rel_otu1)

rel_otu[,1]=rel_otu1[,1]*sam[1,2]

rel_otu[,2]=rel_otu1[,2]*sam[2,2]

rel_otu[,3]=rel_otu1[,3]*sam[3,2]

rel_otu[,4]=rel_otu1[,4]*sam[4,2]

rel_otu[,5]=rel_otu1[,5]*sam[5,2]

rel_otu[,6]=rel_otu1[,6]*sam[6,2]

rel_otu[,7]=rel_otu1[,7]*sam[7,2]

rel_otu[,8]=rel_otu1[,8]*sam[8,2]

rel_otu[,9]=rel_otu1[,9]*sam[9,2]

rel_otu[,10]=rel_otu1[,10]*sam[10,2]

rel_otu[,11]=rel_otu1[,11]*sam[11,2]

rel_otu[,12]=rel_otu1[,12]*sam[12,2]

rel_otu[,13]=rel_otu1[,13]*sam[13,2]

rel_otu[,14]=rel_otu1[,14]*sam[14,2]

rel_otu[,15]=rel_otu1[,15]*sam[15,2]

rel_otu[,16]=rel_otu1[,16]*sam[16,2]

rel_otu[,17]=rel_otu1[,17]*sam[17,2]

rel_otu[,18]=rel_otu1[,18]*sam[18,2]

rel_otu[,19]=rel_otu1[,19]*sam[19,2]

rel_otu[,20]=rel_otu1[,20]*sam[20,2]

rel_otu[,21]=rel_otu1[,21]*sam[21,2]

#head(rel_otu)

xy=rel_otu

xy$Nit1=rowSums(xy[,1:7])

xy$Nit2=rowSums(xy[,8:14])

xy$Nit3=rowSums(xy[,15:21])

head(xy)

#merge

xy$OTU=rownames(xy)

Brate1=merge(B[,c(2,which(colnames(B)=="OTU"))],xy[,c(22,which(colnames(xy)=="OTU"))],by="OTU",all.x=T)

head(Brate1)

colnames(Brate1)=c("OTU","B_1","Nit_1")

Brate1$dNi1=Brate1$Nit_1*(1-exp(-Brate1$B_1*2))/2

Brate2=merge(B[,c(3,which(colnames(B)=="OTU"))],xy[,c(23,which(colnames(xy)=="OTU"))],by="OTU",all.x=T)

head(Brate2)

colnames(Brate2)=c("OTU","B_2","Nit_2")

Brate2$dNi2=Brate2$Nit_2*(1-exp(-Brate2$B_2*2))/2

Brate3=merge(B[,c(4,which(colnames(B)=="OTU"))],xy[,c(24,which(colnames(xy)=="OTU"))],by="OTU",all.x=T)

head(Brate3)

colnames(Brate3)=c("OTU","B_3","Nit_3")

Brate3$dNi3=Brate3$Nit_3*(1-exp(-Brate3$B_3*2))/2

e=merge(Brate1[,c(4,which(colnames(Brate1)=="OTU"))],Brate2[,c(4,which(colnames(Brate2)=="OTU"))],by="OTU",all=T)

Brate_all=merge(e,Brate3[,c(4,which(colnames(Brate3)=="OTU"))],by="OTU",all=T)

head(Brate_all)

Brate_all$N_mean=rowMeans(Brate_all[,2:4])

#

tax <- read.table('../data/taxonomy_clean.txt', sep='\t', header=T, comment.char='', check.names=F)

head(tax)

rownames(tax) <- tax$`OTUID`

tax <- tax[,-1]

tax$OTU=rownames(tax)

ABC=merge(Brate_all,tax[,c(2,3,6,which(colnames(tax)=="OTU"))],by="OTU",all.x=T)

head(ABC)

pro=ABC[which(ABC$Phylum=="Proteobacteria"),]

pro$Phylum1=pro$Class

unpro=ABC[which(ABC$Phylum!="Proteobacteria"),]

unpro$Phylum1=unpro$Phylum

pro=as.data.frame(pro)

unpro=as.data.frame(unpro)

new_phy=rbind(pro,unpro)

head(new_phy)

#

new_phy$Tr="+T-P"###########

write.csv(new_phy,"d_CL0_Brate_+T-P_new_OTU.csv")###########

Phylum=aggregate(new_phy[,c(2:5)],list(new_phy$Phylum1),sum)

Phylum$N_rela=Phylum$N_mean/sum(Phylum$N_mean)

Phylum$Tr="+T-P"###########

write.csv(Phylum,"d_CL0_Brate_+T-P_Phylum.csv")###########

Genus=aggregate(new_phy[,c(2:5)],list(new_phy$Genus),sum)

Genus$N_rela=Genus$N_mean/sum(Genus$N_mean)

Genus$Tr="+T-P"###########

write.csv(Genus,"d_CL0_Brate_+T-P_Genus.csv")###########

#ggplot2

#Phylum1= read.csv("Phylum_rela.csv",header=T, row.names=1)

Phylum1= read.table("clipboard",header=T)

Phylum1

library(ggplot2)

p = ggplot(Phylum1, aes(x=variable, y = N_mean, fill = tax )) +

geom_bar(stat = "identity",position="stack", width=0.9)+

theme(strip.background = element_blank())+

theme_bw() +##

xlab("")+ylab("Birth rate (16S rRNA gene copies/d)")+

ggtitle("zong-birth")+

#theme_classic()+theme(axis.text.x=element_text(angle=45,vjust=1, hjust=1))+#

scale_fill_manual(values =rev(c("I-Acidobacteria"="#EE6677","L-Actinobacteria"="#00FF3B","Z-Alphaproteobacteria"="#882E72","J-Armatimonadetes"="#5289C7","F-Bacteroidetes"="#DC050C","U-Betaproteobacteria"="#994F88","BA-candidate_division_WPS-1"="#90C987","G-Chloroflexi"="#EE8026","M-Deltaproteobacteria"="#BA8DB4","H-Firmicutes"="#66CCEE","N-Gammaproteobacteria"="#AA6F9E","E-Gemmatimonadetes"="#0009AA","O-Ignavibacteriae"="#BBCCEE","K-Nitrospirae"="#F7F056","D-Planctomycetes"="#662506","B-Unassigned"="#7C7C7C","C-Verrucomicrobia"="#FCF7D5","Q-Thaumarchaeota"="#9E8B1E","R-Latescibacteria"="#420073","S-Cyanobacteria"="#1B7837","T-Candidatus_Saccharibacteria"="#0000FF","A-Low_abundance"="#000000"))) +

theme(panel.grid = element_blank(), panel.background = element_rect(color = 'black', fill = 'transparent')) +

theme(legend.title = element_blank())

p

ggsave("birth_zong_dCUless0.pdf",device=cairo_pdf,width=8,height=8)

###
